# Can Smartphone Apps Increase Physical Activity? Systematic Review and Meta-Analysis

**DOI:** 10.2196/12053

**Published:** 2019-03-19

**Authors:** Amelia Romeo, Sarah Edney, Ronald Plotnikoff, Rachel Curtis, Jillian Ryan, Ilea Sanders, Alyson Crozier, Carol Maher

**Affiliations:** 1 School of Health Sciences University of South Australia Adelaide Australia; 2 Alliance for Research in Exercise, Nutrition and Activity School of Health Sciences University of South Australia Adelaide Australia; 3 Priority Research Centre for Physical Activity and Nutrition University of Newcastle Newcastle Australia

**Keywords:** physical activity, smartphone, mobile phone, app, mobile apps, program, health behavior, systematic review, meta-analysis

## Abstract

**Background:**

Smartphone apps are a promising tool for delivering accessible and appealing physical activity interventions. Given the large growth of research in this field, there are now enough studies using the “gold standard” of experimental design—the randomized controlled trial design—and employing objective measurements of physical activity, to support a meta-analysis of these scientifically rigorous studies.

**Objective:**

This systematic review and meta-analysis aimed to determine the effectiveness of smartphone apps for increasing objectively measured physical activity in adults.

**Methods:**

A total of 7 electronic databases (EMBASE, EmCare, MEDLINE, Scopus, Sport Discus, The Cochrane Library, and Web of Science) were searched from 2007 to January 2018. Following the Population, Intervention, Comparator, Outcome and Study Design format, studies were eligible if they were randomized controlled trials involving adults, used a smartphone app as the primary or sole component of the physical activity intervention, used a no- or minimal-intervention control condition, and measured objective physical activity either in the form of moderate-to-vigorous physical activity minutes or steps. Study quality was assessed using a 25-item tool based on the Consolidated Standards of Reporting Trials checklist. A meta-analysis of study effects was conducted using a random effects model approach. Sensitivity analyses were conducted to examine whether intervention effectiveness differed on the basis of intervention length, target behavior (physical activity alone vs physical activity in combination with other health behaviors), or target population (general adult population vs specific health populations).

**Results:**

Following removal of duplicates, a total of 6170 studies were identified from the original database searches. Of these, 9 studies, involving a total of 1740 participants, met eligibility criteria. Of these, 6 studies could be included in a meta-analysis of the effects of physical activity apps on steps per day. In comparison with the control conditions, smartphone apps produced a nonsignificant (*P*=.19) increase in participants’ average steps per day, with a mean difference of 476.75 steps per day (95% CI −229.57 to 1183.07) between groups. Sensitivity analyses suggested that physical activity programs with a duration of less than 3 months were more effective than apps evaluated across more than 3 months (*P*=.01), and that physical activity apps that targeted physical activity in isolation were more effective than apps that targeted physical activity in combination with diet (*P*=.04). Physical activity app effectiveness did not appear to differ on the basis of target population.

**Conclusions:**

This meta-analysis provides modest evidence supporting the effectiveness of smartphone apps to increase physical activity. To date, apps have been most effective in the short term (eg, up to 3 months). Future research is needed to understand the time course of intervention effects and to investigate strategies to sustain intervention effects over time.

## Introduction

### Background

Physical inactivity is the fourth largest behavioral risk factor contributing to ill health and mortality [1]. The number of adults who are physically inactive is on the rise in many countries, influencing the prevalence of noncommunicable diseases and the general health of the global population [2,3]. Current global recommendations on physical activity for health suggest adults (aged 18 to 64 years) participate in at least 150 min of moderate-intensity physical activity or 75 min of vigorous-intensity physical activity per week [4]. Participation in regular physical activity reduces the risk of noncommunicable diseases by improving muscular and cardiorespiratory fitness, functional health, and mental health [2,3,5]. However, currently, 1 in 4 adults globally do not meet physical activity recommendations [2]. As such, finding effective strategies to increase participation in regular physical activity is an essential public health objective.

Smartphone apps are being recognized as a potential and promising approach to increase adherence to physical activity guidelines. Globally, activated mobile phones outnumber citizens [6] with approximately 63% of the global adult population owning at least 1 smartphone in 2017 [7]. Smartphones are inexpensive and allow users to engage with health information technology in any environment and at any time [8]. They are equipped with advanced technological features, most notably their connection to the internet, global positioning system, and inbuilt accelerometers [9], and offer the capacity to create individualized and interactive apps that collect real-time data [10]. These features, as well as the high usage and convenience of smartphones, make them an attractive tool for researchers to deliver physical activity interventions.

Indeed, studies are increasingly using smartphone apps to try to motivate individuals to be physically active. In 2015, a systematic review and meta-analysis of 12 studies using smartphone apps to promote weight loss and increase physical activity found a nonsignificant difference in physical activity between the control group and smartphone intervention group [11]. In 2016, a systematic review of 15 studies, including both qualitative and quantitative research designs, found that smartphone apps can be effective in increasing physical activity although the effect size was modest [12]. However, these reviews are subject to limitations. For example, they only searched a small number of databases, and they included studies with self-reported physical activity data, which are susceptible to bias [13]. In addition, they included studies where smartphone apps were delivered in concert with other intervention elements (eg, face-to-face appointments and podcasts); thus, it is unclear whether intervention effects were truly due to the smartphone app itself, or rather the other intervention elements. Furthermore, the most recent review [12] included studies published until the end of 2015 only. Given the exponential growth in this field [14], the evidence base has expanded considerably since this time. Thus, it is imperative that smartphone intervention research is updated. Finally, these reviews incorporated studies varying widely in research design. Given the large growth of available research in the field, there are now likely to be enough studies using the “gold standard” of experimental design—the randomized controlled trial design—and employing objective measurement of physical activity to support a meta-analysis of scientifically rigorous studies. Examining only studies that meet this stringent methodological inclusion criteria will heighten the trustworthiness of findings.

### Objectives

This systematic review aimed to (1) identify all published randomized controlled trials, which examine the efficacy of physical activity interventions delivered via smartphone apps on increasing objectively measured physical activity and (2) conduct a meta-analysis of these published studies to determine the current state of evidence regarding the effectiveness of smartphone app-based interventions for increasing physical activity in an adult population.

## Methods

### Information Sources and Search Strategy

This systematic review was completed according to the Preferred Reporting Items for Systematic Reviews and Meta-Analyses guidelines [15].

A systematic search was conducted on January 8, 2018, and included 7 electronic databases: EMBASE, EmCare, MEDLINE, Scopus, Sport Discus, The Cochrane Library, and Web of Science. The search strategy was reviewed by an academic librarian before being finalized. Each database was searched individually, and the search strategy for 1 database, EMBASE, is presented in Table 1. In brief, the search strategy combined synonyms for the intervention (mobile phone apps) with synonyms for the outcomes (physical activity and weight loss), and these terms were mapped to MeSH headings where possible. Search results were limited to the English language, humans, and year of publication from 2007 to present (on the basis that the original iPhone was released in 2007). The search strategy for all databases is presented in Multimedia Appendix 1. The reference lists of all eligible studies and of relevant systematic reviews [11,12,16,17] were screened to identify any further studies for inclusion. In addition, 5 prominent researchers in the field were contacted with the list of identified studies and asked to recommend any additional studies that met the inclusion criteria.

**Table 1:** Search strategy as used in EMBASE on January 8, 2018.

Search category	Search terms
Smartphones	Cell phones^a^/Smartphones^a^/Mobile Applications^a^/(“smart phone*” or smartphone* or smart-phone* or “cell*phone*” or “cell-phone*” or “mobile phone*” or “mobile-phone” or “mobile device” or “mobile telephone*” or i*Phone* or android* or iOS or “mobile health” or “mhealth” or “m-health” or app or apps or “mobile application*”)
Physical activity	Exercise^a^/Weight Loss^a^/(“physical activit*” or exercise* or “active living” or walk* or “active transport*” or “leisure activit*” or fitness or “weight loss” or “weight reduction” or “weight maintenance” or “maintaining weight” or “weight management”)
Intervention	(Intervention or program* or trial)
Combined	1 AND 2 AND 3

^a^Denotes MeSH headings.

### Eligibility Criteria

#### Population

Study samples including healthy adults or adults with a specific health condition aged 18 years or over were eligible. Studies with participants characterized by intellectual or marked cognitive impairments or with a severe mobility disorder were excluded from this study as app designs may be specific for limited cognition and mobility.

#### Intervention

Studies that reported a smartphone app as the primary component to the physical activity intervention were included. The smartphone apps were required to explicitly be stand-alone apps available on a smartphone. Apps within other contexts, for example, Facebook apps, were excluded from the study. In addition, studies with interventions that incorporated secondary components such as ongoing personalized support, either in person or over the phone, were excluded.

#### Control or Comparator

Studies that included control groups, which received no intervention or minimal intervention (eg, given only a physical activity goal), were included.

#### Outcomes

For inclusion in the review, studies had to report objectively measured physical activity in the form of either moderate-to-vigorous physical activity (MVPA) min as measured by accelerometry or steps measured either by accelerometry or pedometry at baseline and follow-up.

#### Study Design

Only randomized controlled trials were included in this review. Conference abstracts were excluded.

### Study Selection

Studies were screened for eligibility in duplicate under blinded conditions by 2 independent reviewers (selected from the author list, ie, AR, SE, RC, AC, IS, or JR) as per best practice for systematic reviews [15]. Search results were first screened based on the title and abstract, and any studies that appeared to meet the eligibility criteria, or where eligibility was unclear, progressed to full-text screening. Moreover, 2 independent reviewers then screened the full-text studies to determine eligibility for inclusion in the review. An interrater agreement of 96% (Cohen kappa= 0.53) was reached. Results from each round of screening were compared among reviewers, and conflicts were discussed until consensus was attained.

### Data Collection Process

Pairs of reviewers (either AR, SE, RC, AC, or IS) independently extracted data from each included study using a standardized form developed specifically for this review (see Multimedia Appendix 2). Extracted data included country of study, study participants (population and sample size), study design (app features, description of intervention group and control group, and duration of follow-up), outcome measures (measurement tool and timing), and key study results (mean and SD for MVPA or steps at baseline and follow-up). Where the results were not adequately reported within the study paper, authors were contacted to provide additional data. Where there were discrepancies in data extraction, the author team discussed and rechecked the original study until consensus was reached.

### Risk of Methodological Bias

Risk of bias was assessed using a 25-item tool developed by Maher [18] and based on the Consolidated Standards of Reporting Trials (CONSORT) checklist [19]. Each study was independently scored by 2 reviewers. Items were scored 1 if the study satisfactorily met the criteria, and 0 if the study did not satisfactorily meet the criteria or if the item was not applicable to the study. Disagreements between reviewers were resolved by checking and discussing the original study until consensus was reached. The most common disagreement related to whether items were not present or not applicable to the study, which did not have a large effect on assessment risk of bias as both were scored 0. Studies were also graded using the 2011 Centre for Evidence Based Medicine Levels of Evidence [20].

### Synthesis of Results

The primary outcome measure meta-analyzed in this review was mean change in physical activity either reported in MVPA per day or steps per day. For studies that did not report mean change, it was calculated from the baseline data and follow-up (ie, the end of the intervention) data. Where studies included multiple intervention groups with similar app features, the intervention group with the most app features was included in the meta-analysis.

For the intervention and control group of each study, the mean change in physical activity from baseline to follow-up, SD of the change, and the number of participants were entered into Review Manager software (Version 5.3, The Nordic Cochrane Centre) and used to calculate the mean difference and standardized mean difference between the change in the intervention group and the change in the control group for each study. Where data were available from 3 or more studies, a meta-analysis calculating the combined effects for all studies was performed.

A random effects model approach was used as study heterogeneity was anticipated because of the variance of study populations and intervention designs. The presence of heterogeneity was determined using the I^2^ statistic, which describes the percentage of total variation in study estimates that are a result of heterogeneity [21]. The I^2^ statistic was selected as the preferred measure of variance as it is robust for small sample sizes [21]. The mean difference and standardized mean difference size were interpreted using the Cohen [22] suggestion that 0.2 is considered a *small* effect size, 0.5 a *medium* effect, and 0.8 a *large* effect, with values smaller than 0.2 being *trivial*. Furthermore, 3 sensitivity analyses were performed to further assess the robustness of our study results by excluding either studies with an intervention duration of 3 months or more (or equivalent in weeks per days) or studies with an app designed to increase physical activity as well as other nonphysical activity behaviors or studies designed for disease population groups.

## Results

### Study Selection

**Figure 1:**
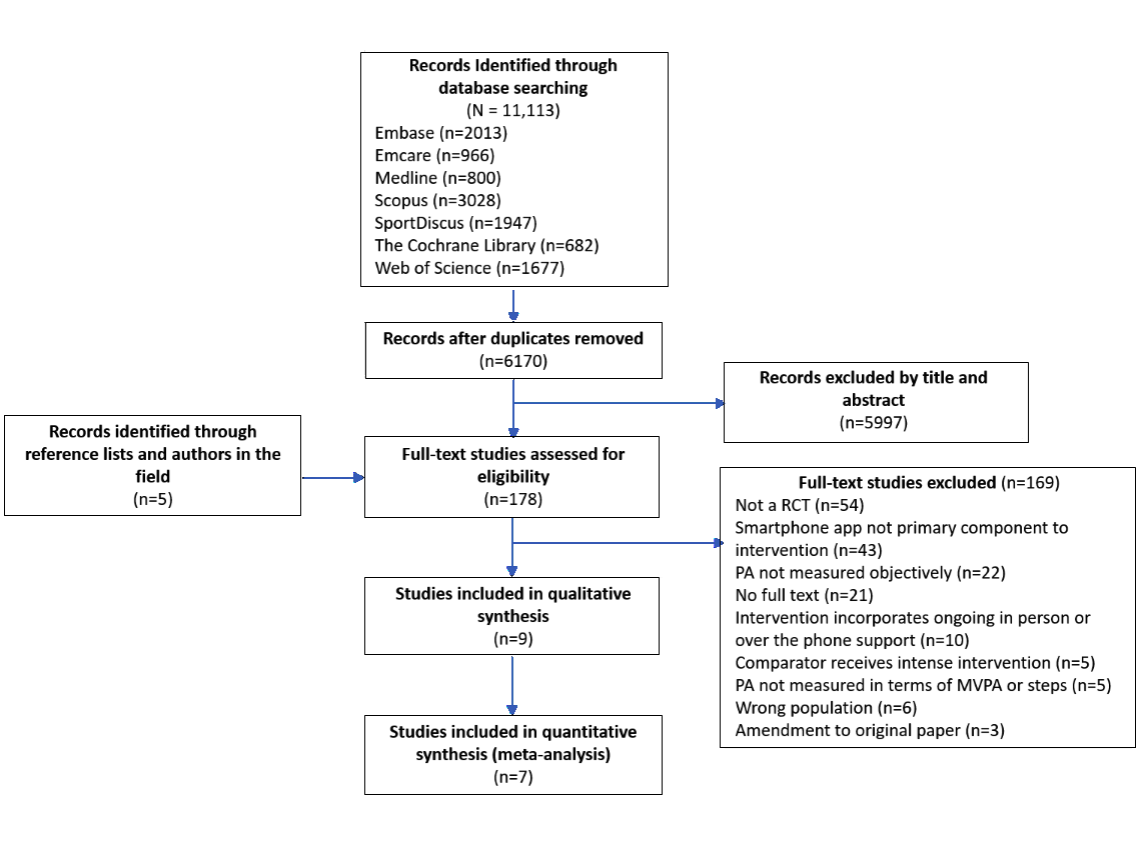
Flowchart for the selection of studies in this meta-analysis. PA: physical activity; MVPA: moderate-to-vigorous physical activity; RCT: randomized controlled trial.

After the removal of duplicates, a total of 6170 studies were identified from the original database search. An additional 5 studies were suggested by leading authors in the field; however, none of these studies met all inclusion criteria for the systematic review. In total, 9 studies were identified to meet the eligibility criteria for inclusion in this review, and 7 of these studies were included in the meta-analysis. Moreover, 1 study [23] had unusable baseline data due to a technical malfunction in their study; this meant that the mean change in physical activity from baseline to follow-up could not be calculated, and the results were unable to be included in the meta-analysis. In addition, despite best efforts to contact the authors of the study to provide further data, 1 study [24] was excluded from the meta-analysis owing to not reporting baseline or follow-up data for the control group. The study selection process is summarized in Figure 1.

### Study Characteristics

All 9 studies reported from this review were randomized controlled trials published in English between 2014 and 2017. The key characteristics of the included studies are presented in Table 2. A total of 3 studies reported being registered with a Clinical Trial Registry [24-26]. Sample sizes ranged from 23 to 833, with a total of 1740 participants across the 9 studies. All studies had a smartphone app designed to increase physical activity as the primary intervention, although the Recio-Rodriguez et al [27] app was also designed to increase both physical activity and adherence to a Mediterranean dietary pattern. Control groups received either no intervention [27-29], a wearable accelerometer only [30,31], or a basic version of a smartphone app [23-26]. Of the 9 studies, 4 reported their app as being based on a recognized behavior-change theory; the social cognitive theory was reported in 3 [24,25,30], and principles of re-enforcement [24], social influencers’ perspective [24], and taxonomy of behavior change [28] were reported in 1 study each. Physical activity data were collected using the smartphones’ in-built accelerometer in 3 of the studies [23,24,26] and using a separate wearable accelerometer in 6 of the studies [25,27-31]. Intervention length ranged from 6 weeks to 6 months. A total of 6 studies reported physical activity in terms of steps per day [26-31], supporting the meta-analysis of this outcome measure. Only 2 studies reported physical activity data in terms of MVPA in sufficient detail to calculate effect sizes, and meta-analysis for this outcome measure could, therefore, not be performed.

### App Features

The intervention apps included a variety of features such as a physical activity performance summary [25,27,29,31], goal setting [24,25,27,28], visual display of goal achievement [24,26,28,29], and motivational prompts [24,30,31]. The only feature common to each of the intervention apps was a visible display of steps or MVPA (see Table 2 for a detailed description of each intervention apps’ features).

### Risk of Bias in Included Studies

All studies were screened for risk of methodological bias using the CONSORT checklist [19] and scores varied from 15 [23,28,30] to 21 out of 25 [24]. Full details of the bias screening are presented in Multimedia Appendix 3. Each study was graded level 2 as a randomized trial using the 2011 Centre for Evidence Based Medicine Levels of Evidence [20]. All studies fulfilled the CONSORT checklist requirements to provide scientific rationale and clearly describe the intervention. The randomization procedure within most of the studies was considered adequate, with allocation concealment mechanisms detailed in all but 1 study [30]. All studies satisfied the CONSORT checklist criteria for providing primary and secondary outcomes and results for each group except for 2 studies [23,24], which were excluded from the meta-analysis.

### Meta-Analysis of Smartphone App–Based Intervention and Steps Per Day

**Figure 2:**
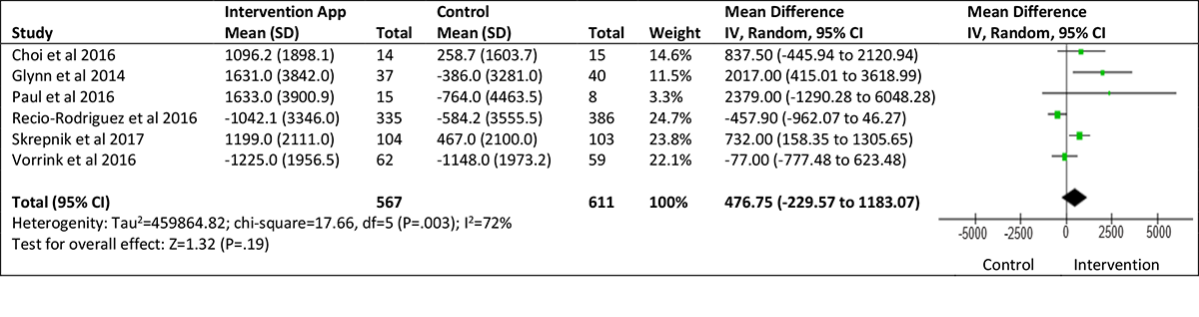
Steps per day mean difference. IV: inverse variance.

**Figure 3:**
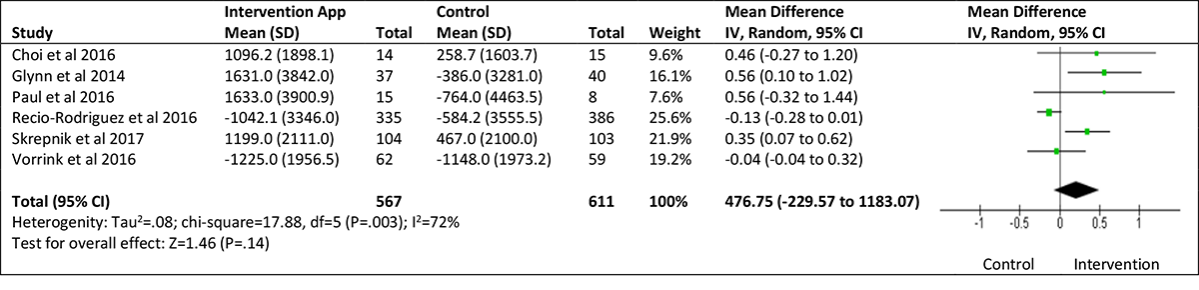
Steps per day standardized mean difference. IV: inverse variance.

A total of 6 studies, involving a total of 1178 participants, reported change in physical activity in terms of steps per day [26-31]. In comparison with the control (the nature of which varied between studies), the intervention conditions showed a nonsignificant (*P*=.19) increase in average steps per day, with a mean difference between groups of 476.75 steps per day (95% CI −229.57 to 1183.07; see Figure 2). The standardized mean difference between the control and intervention groups was small in magnitude and favored the intervention group (0.21; 95% CI −0.07 to 0.50; see Figure 3), yet was not statistically significant (*P*=.14) *.* The impact of heterogeneity within the studies was significant (I^2^=72%), suggesting that 72% of variation across studies was due to heterogeneity rather than chance [32]. Thus, the meta-analysis results should be interpreted with caution.

**Table 2:** Data extraction characteristics of included studies.

Study	Study population/sample size	Smartphone app features	Intervention description	Outcome measures
Choi et al 2016 [28]	Sample size: Total n=30, intervention n=15, control n=15; Population: Pregnant women between 10 and 20 weeks of gestation with a sedentary lifestyle. Age: Mean 33.7 (SD 2.6); Male (%): 0; Country: United States of America; Attrition rate: 3%	Characteristics: Visual display of steps, distance, flights of stairs climbed, and estimated calories expended. A daily message prompt to support PA^a^ was available between 10 am and 7 pm, participants were able to respond to the message and receive feedback. Activity diary available after 7 pm each night. Theory: Social cognitive theory	Focus: Physical activity; Groups: (Intervention) Participants wore a Fitbit and had access to all features within a specifically designed smartphone app. Participants were given the goal of increasing their step count by 10% each week until 8500 steps a day was reached. (Control) Participants wore a Fitbit and were given the goal to increase steps gradually until 8500 steps a day was reached. Additional to app: One face-to-face goal setting session, information provided on healthy diets and recommendations for gestational weight gain, and safety instructions for PA during pregnancy. Duration: 12 weeks; Follow-up post baseline: Weeks 4, 8, and 12.	Primary outcome: Steps per day; Measuring tool: Fitbit Ultra Accelerometer; Secondary outcomes: TV/computer time, self-efficacy, barriers, social support, depressive symptoms, and pregnancy symptoms.
Fanning et al 2017 [30]	Sample size: Total n=116, Group A n=29, Group B n=31, Group C n=26, Group D n=30; Population: Low-active adults; Age: Mean 41.4 (SD 7.6); Male (%): 20; Country: United States of America; Attrition rate: 17%	Characteristics: Tracking of activities, instant feedback on weekly progress, weekly education modules within the app. Guided goal setting module with goals tied within all app features. Points system module with points provided for all in app tasks and accumulated to earn badges. Theory: Social cognitive theory	Focus: Physical activity; Groups: (A) goal setting module and point-based feedback module, (B) goal setting module, (C) point based feedback module, (D) standard app. Additional to app: Text messages reminding participants to goal set and be active and track activities. Support emails at the beginning of each week. Counseling on SMART^c^ goal setting (groups A and B). Duration: 12 weeks; Follow-up post baseline: 12 weeks	Primary outcome: Mean daily minutes of MVPA^b^. Measuring tool: Actigraph accelerometers (model GT1 M or newer); Secondary outcomes: self-efficacy, perceived barriers, outcome expectations, goals, use, and usability.
Glynn et al 2014 [31]	Sample size: Total n=90, intervention n=45, control n=45; Population: Existing Android smartphone users; Age: Mean 44.1 (SD 11.5); Male (%): 36; Country: Ireland; Attrition rate: 14%	Characteristics: Automatic feedback and tracking of step count and calories burned, visually appealing display of step count history, and goal achievement. Theory: N/A^d^	Focus: Physical activity; Groups: (Intervention) Access to app, instruction to interact with app and goal of 10,000 steps per day; (control) goal of 30 min activity per day, access to app without visible tracking or display. Additional to app: Physical activity goals, information on the benefits of exercise, and physical activity promotion brochure. Duration: 8 weeks; Follow-up post baseline: Weeks 2-8	Primary outcome: Steps per day; Measuring tool: Accelerometer within smartphone and share data function of the app. Secondary outcomes: Mean systolic blood pressure, mean diastolic blood pressure, mean resting heart rate, weight and body mass index, mental health quality of life, and quality of life.
Harries et al 2016 [23]	Sample size: Total n=165, intervention (group 1) n=55, intervention (group 2) n=55, control n=55; Population: Males with an existing mobile phone contract; Age range: 22-40 years; Male (%): 100; Country: United Kingdom; Attrition rate: 8%	Characteristics: Step count, steps taken, miles walked, and calories burned for the day and previous week viewable. Social feedback group were able to view their average step count in comparison with other users’ average step counts. Theory: N/A	Focus: Physical activity; Groups: (Intervention group 1) App with feedback on step counts; (intervention group 2) App with feedback on step counts plus social comparison; (control) basic app, no feedback or social features. Additional to app: Standardized text-messages in the first 2 weeks to remind participants to carry their phones in their pockets. Intervention groups received weekly messages to encourage them to walk more. Duration: 6 weeks; Follow-up post baseline: N/A	Primary outcome: Steps per day; Measuring tool: Accelerometer within mobile phone. Secondary outcomes: N/A
King et al 2016 [24]	Sample size: Total n=89, affect group n=22, analytic group n=21, social group n=22, control n=24; Population: Underactive adults aged 45+; Age: Mean 60.0 (SD 9.3); Male (%): 24.7; Country: United States of America; Attrition rate: 6%	Characteristics: (Analytic app) goal-setting, behavioral feedback, tips promoting behavior change, and problem-solving strategies with 2 colorful meters showing progress toward MVPA and sedentary behavior goals; (Social app) social support for behavior change, “just-in-time” social normative feedback, modelling of behaviors by others using avatars on the display, and group-based collaboration and competition “virtual teams”; (affect app) utilized an avatar bird to mirror how active or sedentary the user was throughout the day. The bird avatar changed position, posture, and movement depending on how active/inactive user was. Users received “rewards” as PA levels increased; Theory: (Analytic) social cognitive theory; (social) social influence perspectives; (affect) principles of reinforcement scheduling and attachment, and nurturance motives.	Focus: Physical activity and sedentary behavior; Groups: (analytic) Access to analytic app; (social) access to social app; (affect) access to affect app; (control) access to commercially accessible nonphysical activity dietary app (calorific). Additional to app: Initial 1-hour training on how to use the smartphone app. Duration: 8 weeks; Follow-up post baseline: Weeks 2-8	Primary outcome: Mean daily mins of MVPA; Measuring tool: Accelerometer within smartphone. Secondary outcomes: estimated minutes of sedentary time, self-reported sociological momentary assessment of daily brisk walking and sitting time.
Paul et al 2016 [26]	Sample size: Total n=23, intervention n=15, control n=8; Population: Stroke survivors who have had a single unilateral stroke and can walk independently with or without an aid; Age: Mean 55.8 (SD 10.7); Male (%): 48; Country: United Kingdom (Scotland); Attrition rate: 4%	Characteristics: Step count, goal-setting, planning, monitoring, and feedback, as well as rewards and social facilitation. Within the app, participants are represented by a fish within a fish tank. The fish swims and blows bubbles when the participant is active (which other participants can see). Fish fins and tail grow when targets are achieved. Theory: Taxonomy of behavior change	Focus: Physical activity; Groups: (Intervention) Received a smartphone with the STARFISH APP, individual step goals which increased by 5% each week if participants reached their step goal on 5 of 7 days. Individual and group rewards provided when goals achieved; (control) usual care after stroke (no active rehabilitation); Additional to app: (Intervention) face-to-face at week 3 to discuss progress with Clinical Research Facility; Duration: 6 weeks; Follow-up post baseline: 6 Weeks	Primary outcome: Steps per day; Measuring tool: ActivPAL accelerometer; Secondary outcomes: Sedentary time, heart rate, blood pressure, body mass index, fatigue severity scale, instrumental activity of daily living scale, 10-meter walk test, stroke specific quality of life scale, and psychological general well-being index.
Recio-Rodriguez et al 2016 [25]	Sample Size: Total n=833, intervention n=415, control n=418; Population: Selected from the Multicenter Assessment of Experimental Program Promoting Physical Activity; Age: intervention mean 51.4 (SD 12.1); control mean 52.3 (SD 12); Male (%): intervention 40; control 36; Country: Spain; Attrition rate: 16%	Characteristics: Automatic feedback from accelerometer, goal-setting, and self-monitoring/entry of food intake. End of each day the app reported food intake, PA performance summary, and a balance of ingested and spent calories. This information was used by the app to generate a recommended plan for the following day to improve eating habits and increase PA. Theory: N/A	Focus: Physical activity and Mediterranean diet; Groups: (Intervention) Training and access to mobile phone app and initial standardized counseling in PA and the Mediterranean diet; (control) initial standardized counseling in PA and the Mediterranean diet. Additional to app: Initial counseling session on PA and the Mediterranean diet and print out of support materials. Duration: 3 months; Follow-up post baseline: 3 months	Primary outcome: MVPA and Steps per day; Measuring tool: Actigraph GT3X accelerometer; Secondary outcomes: Adherence to the Mediterranean diet, blood pressure, waist circumference, body mass index, and laboratory parameters.
Skrepnik et al 2017 [29]	Sample size: Total n=211, intervention n=107, control n=104; Population: Adults who have had unilateral knee OA^e^ and have been suitable for treatment with Hylan G-F 20. Age: mean 62.6 (SD 9.4); Male (%): 49; Country: United States of America; Attrition rate: 2%	Characteristics: The OA GO app provided motivational messages and requested participants enter mood and pain data once a day. The app displayed daily step count, calories burned, and sleep. Daily and monthly cumulative activity trends were available to view. Theory: N/A	Focus: Physical activity; Groups: (Intervention) Jawbone UP activity tracker and access to OA GO mobile app; (control) Jawbone UP activity tracker. Additional to app: All patients received a single 6 ml injection of Hylan G-F 20 and regular follow-ups as per standard of care. Duration: 90 days; Follow-up post baseline: 90 days	Primary outcome: Steps per day; Measuring tool: Jawbone UP 24 activity tracker; Secondary outcomes: Mean percentage change from baseline in the 6-min walk test, patient and physician satisfaction with treatment, percentage change in Patient Activation Measure (PAM)-13 questionnaire score, percentage change in sleep captured by the wearable activity monitor (light, sound, and duration of sleep), and Visual Analog Mood Scale.
Vorrink et al 2016 [27]	Sample size: Total n=183, intervention n=102, control n=81; Population: Physiotherapy patients with COPD, GOLD stage 2 or 3 who had completed a pulmonary rehabilitation program of 3 months. Age: intervention mean 62.0 (SD 9.0); control mean 63.0 (SD 8.0); Male (%): 50; Country: United States of America; Attrition rate: 34%	Characteristics: App displayed physical activity in real-time in quantitative and qualitative form. It displays the total number of steps taken each day relative to the daily goal and offers advice on physical activity progress. Theory: N/A	Focus: Physical activity; Groups: (Intervention) smartphone and app with physical activity goals and automated persuasive messages; (control) usual care. Additional to app: Physiotherapists could monitor patients and adjust their goals or send messages through a website. Duration: 12 months; Follow-up post baseline: 12 months	Primary outcome: Steps per day; Measuring tool: SenseWear Pro or MF-SW mini armband accelerometers. Secondary outcomes: Average METS^f^, 6-min walking distance, dyspnea, fatigue, emotional function, mastery, and body mass index

^a^PA: physical activity.

^b^MVPA: moderate-to-vigorous physical activity.

^c^SMART: Specific, Measurable, Achievable, Realistic, and Timely.

^d^N/A: not applicable.

^e^OA: osteoarthritis.

^f^MET: metabolic equivalent of task.

### Effects of Smartphone App–Based Intervention on Moderate-to-Vigorous Physical Activity Per Day

Time spent in MVPA was reported in 2 studies [25,27] that included a combined total of 732 participants. Both studies reported a nonsignificant trend for daily MVPA minutes to decrease in the intervention group (see Table 3). However, the effect sizes were less than 0.2 (*trivial* [22]) in magnitude as they were in the order of 2 to 3 min per day difference, or an effect size in the order of -0.1 (*trivial* [22]). Due to the small sample (two studies), meta-analysis was not performed.

### Sensitivity Analyses

Sensitivity analyses were conducted to determine whether the meta-analysis results were consistent under different conditions. Figures for the results of the sensitivity analyses are available in Multimedia Appendix 4. In the first sensitivity analysis, studies with an intervention length of up to 3 months were included (ie, the studies with an intervention length more than or equal to 3 months were excluded) [27,29-31]. The meta-analysis results suggested physical activity apps significantly increased steps per day by 2074.96 steps per day (95% CI 606.80 to 3543.11, standardized mean difference 0.56, 95% CI 0.16 to 0.97, *P*=.01).

**Table 3:** Moderate-to-vigorous physical activity effect size for mean difference and standardized mean difference.

Study	Intervention app		Control		Mean difference		Standardized mean difference	
	Mean (SD)	N	Mean (SD)	N	Weight (%)	IV^a^, Random, 95% CI	Weight (%)	IV, Random (95% CI)
Fanning et al 2017 [30]	11.9 (25.7)	26	14.1 (24.4)	27	9.0	−2.16 (−15.68 to 11.36)	7.2	−0.08 (−0.06 to 0.45)
Recio-Rodriguez et al 2016 [25]	−7.9 (27.1)	335	−4.3 (29.2)	344	91	−3.16 (−7.85 to 0.63)	92.8	−0.13 (−0.28 to 0.02)

^a^IV: inverse variance.

A second sensitivity analysis examined whether effects for studies that targeted physical activity alone (ie, the one study that intervened on physical activity and diet together was excluded [27]). The meta-analysis results suggested that physical activity apps increased physical activity by 716.86 steps per day (95% CI 38.37 to 1395.36, *P*=.04) or by a standardized mean difference of 0.31 (95% CI 0.07 to 0; *P*=.01).

A final sensitivity analysis examined whether the effects of physical activity apps were consistent for apps targeting a general adult population or populations with specific health conditions (eg, stroke survivors). In both cases, results were consistent with the main meta-analysis; that is, there was a nonsignificant trend for improvement in daily steps in both general adult populations (+649.54 steps per day; 95% CI −822.66 to 2121.74; standardized mean difference 0.24; 95% CI −0.30 to 0.78; *P*=.24) and in populations with specific health conditions (+438.36 steps per day; 95% CI −335.94 to 1212.67; standardized mean difference 0.22; 95% CI −0.09 to 0.53; *P*=.17).

## Discussion

### Principal Findings

This meta-analysis suggests that app-based physical activity interventions have a nonsignificant, positive influence on objectively measured physical activity. However, sensitivity analyses suggest that effects differ based on study parameters. In particular, there is evidence that smartphone apps have a significant positive effect on physical activity when used over a short-term period (ie, less than 3 months) and where apps target physical activity alone, rather than physical activity in combination with other health behaviors.

To the best of our knowledge, this is the first meta-analysis to establish the effectiveness of app-based physical activity interventions at increasing objectively measured physical activity. Only 1 meta-analysis of app-based physical activity interventions has been published [11], which differs from this study in that it reports on weight loss and subjective physical activity data from both nonrandomized and randomized controlled trials published through to August 2015. Despite these differences, our findings are broadly consistent with both studies finding a nonsignificant increase in physical activity in comparison with control. The other recent systematic review found smartphone apps to have a modest effect on physical activity and noted the limited number of randomized controlled trials that were available at that time to test the efficacy of smartphone apps at increasing physical activity [12]. Our study confirmed that the number of randomized controlled trials evaluating efficacy using objective measurements of physical activity has increased, yet is still limited.

Sensitivity analyses suggested that app-based physical activity interventions were effective when the intervention duration was 3 months or less, compared with longer interventions. This is consistent with findings from 2 other studies [33,34] who similarly reported that physical activity apps appear to be most effective with durations longer than 1 month and 8 weeks, respectively. Taken together, these findings suggest that intervention effects appear to peak within the first couple of months of intervention commencement, and dwindle over time. This raises the possibility that studies with an intervention duration of 3 months or more and those that take their first follow-up assessment at 3 months or later may, in fact, be failing to capture intervention effects, which may have peaked and already started to fade by the time assessments are performed.

That physical activity apps may be most effective in the short term is also consistent with previous studies of engagement with technology-based physical activity interventions, which typically find that engagement declines over time, and this decline corresponds with tapering of intervention effectiveness [18,34-37]. Engagement decline is especially prominent in smartphone-based interventions, as their design precludes human support and supportive accountability [37]. In Recio-Rodriguez et al’s [27] trial, just over half of the study population engaged with the app beyond 8 weeks, and the results of the trial favored the control group. Interestingly, the participants in that study who continued to engage with the app for more than 8 weeks actually showed a net increase in MVPA of a mean 44.0 min per week (95% CI 2.1 to 86.0) favoring the intervention group, but this effect was washed out by poor results for the participants who were no longer engaging. Exposure to the intervention is imperative for the intervention to have effect and exposure occurs through participant engagement with the app [36,38]. Thus, these results underscore the notion that ongoing participant engagement with an app is important for intervention effectiveness [36].

Results from the sensitivity analysis suggest that it may be more effective to intervene in physical activity alone rather than in combination with other health behaviors. Note that this interpretation can only be made with caution, given that only 1 study used a multibehavior approach. However, this interpretation is consistent with another recent review that also found apps targeting single health behaviors appear to produce larger improvements than those using a multibehavior approach [34]. In contrast, a recent case study examining the effectiveness of physical activity apps from the perspective of users suggests apps which combined physical activity and diet components were perceived by users to be more effective than apps with physical activity components alone [33]. User preference for combined physical activity and diet apps is likely owing to the large amount of feedback participants received [33] and the correlation between receiving feedback and motivation to engage with health behaviors [39].

### Strengths and Limitations

The stringent inclusion criteria are a key strength of this study, positioning it as the first meta-analysis examining the effectiveness of smartphone app–based interventions on objectively measured physical activity. Maturation of this fast-evolving field allowed us to limit the review to randomized controlled trial methodologies and objective outcomes, heightening the trustworthiness of findings and reducing the likelihood of results being influenced by recall or response bias. In addition, an extensive search of 7 databases was undertaken to reduce the risk of publication bias.

This study is also subject to limitations. Only a relatively small number of studies meeting our strict inclusion criteria were identified and, of these, most had small sample sizes and short intervention lengths. As a result, the confidence intervals for the effect size estimates were quite large, which may have impeded the meta-analysis from determining a significant effect. Despite our strict inclusion criteria, studies were still highly diverse in terms of intervention format, target populations, and study design elements, and heterogeneity scores suggest that the results do not reflect the same pool of data. In particular, some control groups received a minimal intervention [25,26,30,31], which potentially diluted the intervention effect. In addition, although we attempted to focus solely on smartphone apps, some of the included studies included other elements (eg, activity trackers), which in themselves may alter physical activity. This made it impossible to isolate the effects of the mobile phone app component of these interventions. It is acknowledged that although randomized controlled trials are considered the gold-standard experimental design, including only randomized controlled trials in our search criteria excludes studies conducted within ecologically valid designs. As a result, it is possible that our results, based on randomized controlled trials, may differ from those produced by more *real-life* study designs, impeding our ability to make comment on the generalizability of our results to real-world settings. It is also acknowledged that in addition to randomized controlled trials, other study designs with less positivistic assumptions will play an important role in progressing this scientific field [40].

### Future Recommendations

This meta-analysis highlights that relatively few high-quality studies have been conducted examining the effectiveness of physical activity smartphone interventions. Future studies should describe their intervention and app features with adequate detail so that results are reproducible, can be learnt from, and advance this field of research.

Future research should be directed toward enhancing understanding of the time course of intervention effects. In particular, increased understanding of the timepoint at which peak effect size is reached, the timepoint at which user engagement decreases, and the factors that underpin these phenomena are required. This may involve future studies with longer follow-up periods and with outcome measurements taken at more regular and frequent timepoints. The relatively short-term nature of positive effects suggests that additional efforts are required to design app features which help sustain user engagement with the app over time, for example, perhaps through modules, unlockable content, and rewards. Sustaining user engagement is particularly important for smartphone-based interventions due to the absence of human support and supportive accountability [37]. Previous research determined the ease of use, function, feedback, tailored information, ability to personalize design, and design-aesthetic as highly ranked engagement strategies [38]. It will be useful for future app designs to incorporate these long-term engagement strategies, as increased exposure to the intervention is suggested to lead to larger, longer lasting effects [35]. Further research is required to confirm or refute our finding that intervening on 1 health behavior could be more effective than interventions targeting multiple health behaviors.

Key recommendations include the following:

1. Research utilizing randomized controlled trial design, in addition to more ecologically valid designs, is required to progress the field.
2. Studies should be designed to improve our understanding of the time course of intervention effects. This could be achieved through more regular assessments throughout the intervention period, rather than the current preponderance for few widely spaced assessments.
3. Strategies to boost ongoing engagement are required to aid sustainable effectiveness.
4. Further research is required to understand whether it is better to target physical activity as a single behavior or in concert with other health behaviors.

### Conclusions

This is the first systematic review and meta-analysis of smartphone apps for increasing objectively measured physical activity. Results suggest that such apps lead to a nonsignificant increase in objectively measured physical activity, though effectiveness appears greater in physical activity apps when used in the short term and when the apps target physical activity alone. Overall, the meta-analysis offers modest support for the effectiveness of smartphone physical activity apps.

## Appendix

Multimedia Appendix 1 Search strategy for all databases.

Multimedia Appendix 2 Blank data extraction form.

Multimedia Appendix 3 Risk of bias based on Consolidated Standards of Reporting Trials checklist.

Multimedia Appendix 4 Sensitivity analysis results.

## Abbreviations

CONSORT: Consolidated Standards of Reporting Trials
MET: metabolic equivalent of task
MVPA: moderate-to-vigorous physical activity
OA: osteoarthritis
PA: physical activity
SMART: Specific, Measurable, Achievable, Realistic, and Timely
