# Supplementary material for: Can Smartphone Apps Increase Physical Activity? Systematic Review and Meta-Analysis
Source: J Med Internet Res. 2019 Mar 19;21(3):e12053. doi: 10.2196/12053 (PMC6444212; doi:10.2196/12053)
Supplement: Multimedia Appendix 1 [file jmir_v21i3e12053_app1.pdf]

Search terms as used in Embase, Medline and Emcare

Search Category	Search Terms
1. Smartphones	Cell phones/<br>Smartphones/<br>Mobile Applications/<br>(“smart phone*” or smartphone* or smart-phone* or “cell*phone*” or “cell-phone*” or “mobile phone*” or “mobile-phone” or “mobile device” or “mobile telephone*” or i*Phone* or android* or iOS or “mobile health” or “mhealth” or “m-health” or app or apps or “mobile application*”)
2. Physical Activity	Exercise/<br>Weight Loss/<br>(“physical activit*” or exercise* or “active living” or walk* or “active transport*” or “leisure activit*” or fitness or “weight loss” or “weight reduction” or “weight maintenance” or “maintaining weight” or “weight management”)
3. Intervention	(Intervention or program* or trial)
4. Combined	1 AND 2 AND 3

Search terms as used in Scopus, SportDiscus, Web of Science and The Cochrane Library

Search Category	Search Terms
1. Smartphones	(“smart phone*” or smartphone* or smart-phone* or “cell*phone*” or “cell-phone*” or “mobile phone*” or “mobile-phone” or “mobile device” or “mobile telephone*” or i*Phone* or android* or iOS or “mobile health” or “mhealth” or “m-health” or app or apps or “mobile application*”)
2. Physical Activity	(“physical activit*” or exercise* or “active living” or walk* or “active transport*” or “leisure activit*” or fitness or “weight loss” or “weight reduction” or “weight maintenance” or “maintaining weight” or “weight management”)
3. Intervention	(Intervention or program* or trial)
4. Combined	1 AND 2 AND 3

Search results in each database were limited to the English Language, humans and year of publication from 2007 to present.
