# Supplementary material for: Can Smartphone Apps Increase Physical Activity? Systematic Review and Meta-Analysis
Source: J Med Internet Res. 2019 Mar 19;21(3):e12053. doi: 10.2196/12053 (PMC6444212; doi:10.2196/12053)
Supplement: Multimedia Appendix 3 [file jmir_v21i3e12053_app3.pdf]

Multimedia Appendix 2: Risk of Bias Tool – Based on CONSORT Checklist

Item No	Section/ Topic and Checklist item	Choi et. al. 2016.	Fanning et. al 2017.	Glynn et. al. 2014	Harries et. al. 2016.	King et. al 2016.	Paul et. al. 2016.	Recio-Rodriguez et. al. 2016.	Skrepnik et. al. 2017.	Vorrink et. al. 2016.
	**Title and Abstract**									
1a	Identification as a randomised trial in the title	1	1	1	1	1	0	1	1	0
1b	Structured summary of trial design, methods, results, and conclusions (for specific guidance see CONSORT for abstracts)	1	1	1	1	1	1	1	1	0
	**Introduction**									
	**Background and Objectives**									
2a	Scientific background and explanation of rationale	1	1	1	1	1	1	1	1	1
2b	Specific objectives or hypotheses	1	1	1	1	1	1	1	1	1
	**Methods**									
	**Trial Design**									
3a	Description of trial design (such as parallel, factorial) including allocation ratio	0	1	1	0	1	1	1	1	1
3b	Important changes to methods after trial commencement (such as eligibility criteria), with reasons	0	0	1	0	0	0	0	0	0
	**Participants**									
4a	Eligibility criteria for participants	1	1	1	1	1	1	1	1	1
4b	Settings and locations where the data were collected	1	1	1	1	0	1	1	1	1
	**Intervention**									
5	The interventions for each group with sufficient details to allow replication, including how and when they were actually administered	1	1	1	1	1	1	1	1	1
	**Outcomes**									
6a	Completely defined pre-specified primary and secondary outcome measures, including how and when they were assessed	1	1	1	1	1	1	1	1	1
6b	Any changes to trial outcomes after the trial commenced, with reasons	0	0	0	1	0	0	0	0	0
	**Sample Size**									
7a	How sample size was determined	0	1	1	1	1	1	1	1	1

Multimedia Appendix 2: Risk of Bias Tool – Based on CONSORT Checklist

7b	When applicable, explanation of any interim analyses and stopping guidelines	0	0	0	0	0	0	0	0	0
	**Randomisation: Sequence Generation**									
8a	Method used to generate the random allocation sequence	1	1	1	1	1	1	1	1	1
8b	Type of randomisation; details of any restriction (such as blocking and block size)	1	1	1	0	0	1	1	0	1
	**Allocation Concealment Mechanism**									
9	Mechanism used to implement the random allocation sequence (such as sequentially numbered containers), describing any steps taken to conceal the sequence until interventions were assigned	0	1	1	1	1	1	1	1	1
	**Implementation**									
10	Who generated the random allocation sequence, who enrolled participants, and who assigned participants to interventions	0	0	1	0	1	0	1	1	0
	**Blinding**									
11a	If done, who was blinded after assignment to interventions (for example, participants, care providers, those assessing outcomes) and how	0	1	1	1	1	0	1	0	1
11b	If relevant, description of the similarity of interventions	0	0		1	1	0	0	1	1
	**Statistical Methods**									
12a	Statistical methods used to compare groups for primary and secondary outcomes	1	1	1	1	1	1	1	1	1
12b	Methods for additional analyses, such as subgroup analyses and adjusted analyses	0	1	1	0	1	0	1	0	1
	**Results**									
	**Participant Flow**									
13a	For each group, the numbers of participants who were randomly assigned, received intended treatment, and were analysed for the primary outcome	1	1	1	1	1	1	1	1	1

Multimedia Appendix 2: Risk of Bias Tool – Based on CONSORT Checklist

13b	For each group, losses and exclusions after randomisation, together with reasons	1	1	1	1	1	1	1	1	1
	**Recruitment**									
14a	Dates defining the periods of recruitment and follow-up	1	1	1	1	1	0	1	0	0
14b	Why the trial ended or was stopped	0	0	0	0	0	0	0	1	0
	**Baseline Data**									
15	A table showing baseline demographic and clinical characteristics for each group	1	1	1	1	1	1	1	1	1
	**Numbers Analysed**									
16	For each group, number of participants (denominator) included in each analysis and whether the analysis was by original assigned groups	1	1	1	1	1	1	1	1	1
	**Outcomes and Estimation**									
17a	For each primary and secondary outcome, results for each group, and the estimated effect size and its precision (such as 95% confidence interval)	1	1	1	0	0	1	1	1	1
17b	For binary outcomes, presentation of both absolute and relative effect sizes is recommended	0	0	0	0	0	0	0	0	1
	**Ancillary Analyses**									
18	Results of any other analyses performed, including subgroup analyses and adjusted analyses, distinguishing pre-specified from exploratory	0	1	0	0	1	0	1	0	0
	**Harms**									
19	All-important harms or unintended effects in each group (for specific guidance see CONSORT for harms)	1	0	0	0	1	0	0	1	0
	**Discussion**									
	**Limitations**									
20	Trial limitations, addressing sources of potential bias, imprecision, and, if relevant, multiplicity of analyses	1	1	1	1	1	1	1	1	1
	**Generalisability**									
21	Generalisability (external validity, applicability) of the trial findings	1	1	1	0	1	1	1	1	1

Multimedia Appendix 2: Risk of Bias Tool – Based on CONSORT Checklist

	**Interpretation**									
22	Interpretation consistent with results, balancing benefits and harms, and considering other relevant evidence	1	1	1	1	1	1	1	1	1
	**Other Information**									
	**Registration**									
23	Registration number and name of trial registry	1	1	1	0	1	0	0	0	0
	**Protocol**									
24	Where the full trial protocol can be accessed, if available	0	0	1	0	1	0	0	0	0
	**Funding**									
25	Sources of funding and other support (such as supply of drugs), role of funders	1	0	1	1	1	1	1	1	1
	**TOTAL**	**15**	**18**	**20.5**	**15**	**21**	**15**	**19**	**18**	**16.5**
	**CEBM Level of Evidence**									
	**Level 1** Systematic review of randomized trials or n-of-1 trials									
	**Level 2** Randomized trial or observational study with dramatic effect	2	2	2	2	2	2	2	2	2
	**Level 3** Non-randomized controlled cohort/follow-up study**									
	**Level 4** Case-series, case-control studies, or historically controlled studies									
	**Level 5** Mechanism-based reasoning									
