# Supplementary material for: Can Smartphone Apps Increase Physical Activity? Systematic Review and Meta-Analysis
Source: J Med Internet Res. 2019 Mar 19;21(3):e12053. doi: 10.2196/12053 (PMC6444212; doi:10.2196/12053)

Sensitivity Analysis Results

Figure 1: Standardised mean difference for studies with an intervention length of up to three months.

Study or Subgroup	Intervention App Mean	SD	Total	Control Mean	SD	Total	Weight	Std. Mean Difference IV, Random, 95% CI
Choi 2016	1,096.2	1,898.1	14	258.7	1,603.7	15	0.0%	0.46 [-0.27, 1.20]
Glynn 2014	1,631	3,842	37	-386	3,281	40	78.7%	0.56 [0.10, 1.02]
Paul 2016	1,633	3,900.87	15	-746	4,463.51	8	21.3%	0.56 [-0.32, 1.44]
Recio-Rodriguez 2016 (Steps)	-1,042.1	3,346	335	-584.2	3,555.5	386	0.0%	-0.13 [-0.28, 0.01]
Skrepnik 2017	1,199	2,111	104	467	2,100	103	0.0%	0.35 [0.07, 0.62]
Vorrink 2016	-1,225	1,956.46	62	-1,148	1,973.21	59	0.0%	-0.04 [-0.40, 0.32]
**Total (95% CI)**			**52**			**48**	**100.0%**	**0.56 [0.16, 0.97]**

Heterogeneity: Tau² = 0.00; Chi² = 0.00, df = 1 (P = 1.00); I² = 0%
Test for overall effect: Z = 2.71 (P = 0.007)

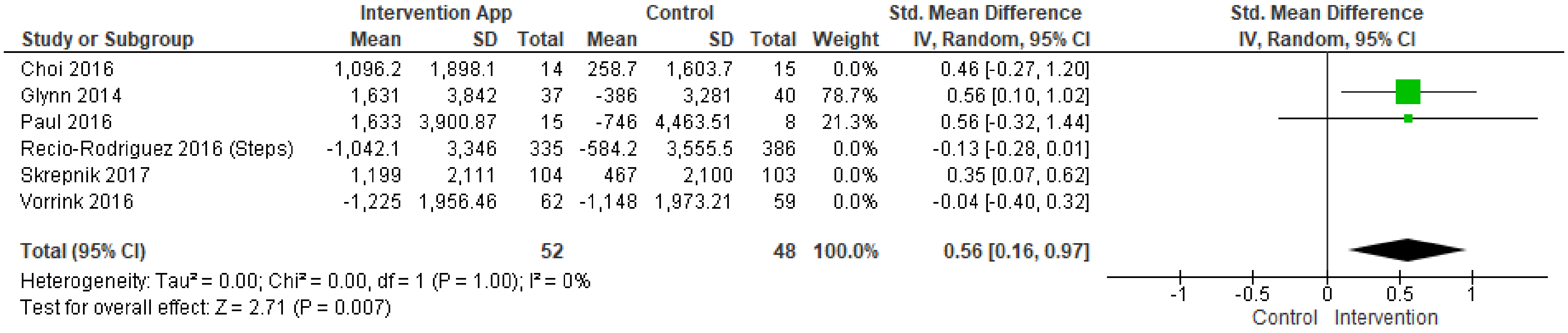

Figure 2: Standardised mean difference for studies that targeted physical activity alone.

Study or Subgroup	Intervention App Mean	SD	Total	Control Mean	SD	Total	Weight	Std. Mean Difference IV, Random, 95% CI
Choi 2016	1,096.2	1,898.1	14	258.7	1,603.7	15	8.8%	0.46 [-0.27, 1.20]
Glynn 2014	1,631	3,842	37	-386	3,281	40	19.5%	0.56 [0.10, 1.02]
Paul 2016	1,633	3,900.87	15	-746	4,463.51	8	6.5%	0.56 [-0.32, 1.44]
Recio-Rodriguez 2016 (Steps)	-1,042.1	3,346	335	-584.2	3,555.5	386	0.0%	-0.13 [-0.28, 0.01]
Skrepnik 2017	1,199	2,111	104	467	2,100	103	37.6%	0.35 [0.07, 0.62]
Vorrink 2016	-1,225	1,956.46	62	-1,148	1,973.21	59	27.6%	-0.04 [-0.40, 0.32]
**Total (95% CI)**			**232**			**225**	**100.0%**	**0.31 [0.07, 0.54]**

Heterogeneity: Tau² = 0.02; Chi² = 5.36, df = 4 (P = 0.25); I² = 25%
Test for overall effect: Z = 2.58 (P = 0.010)

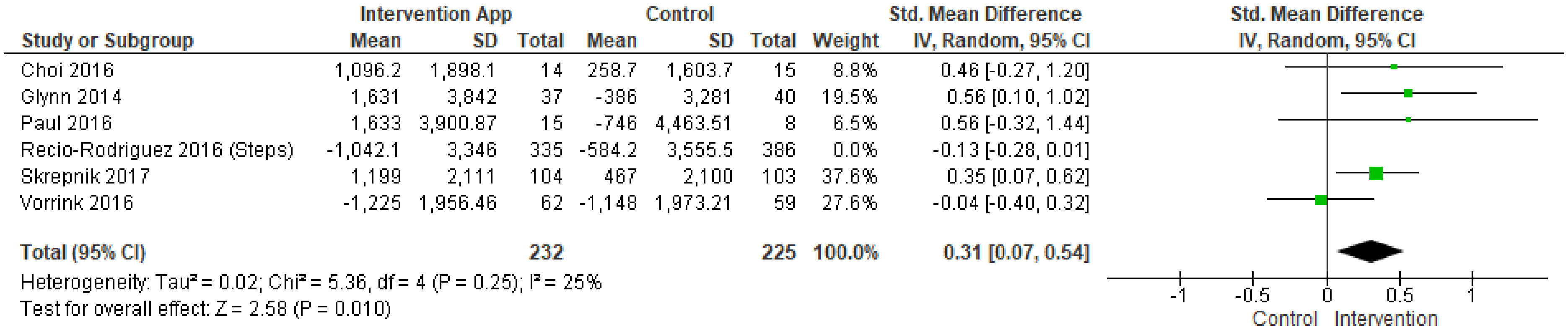

Figure 3: Standardised mean difference for apps targeting a general adult population.

Study or Subgroup	Intervention App Mean	Intervention App SD	Intervention App Total	Control Mean	Control SD	Control Total	Weight	Std. Mean Difference IV, Random, 95% CI
Choi 2016	1,096.2	1,898.1	14	258.7	1,603.7	15	24.1%	0.46 [-0.27, 1.20]
Glynn 2014	1,631	3,842	37	-386	3,281	40	33.4%	0.56 [0.10, 1.02]
Paul 2016	1,633	3,900.87	15	-746	4,463.51	8	0.0%	0.56 [-0.32, 1.44]
Recio-Rodriguez 2016 (Steps)	-1,042.1	3,346	335	-584.2	3,555.5	386	42.5%	-0.13 [-0.28, 0.01]
Skrepnik 2017	1,199	2,111	104	467	2,100	103	0.0%	0.35 [0.07, 0.62]
Vorrink 2016	-1,225	1,956.46	62	-1,148	1,973.21	59	0.0%	-0.04 [-0.40, 0.32]
**Total (95% CI)**			**386**			**441**	**100.0%**	**0.24 [-0.30, 0.78]**

Heterogeneity: $Tau^2 = 0.17$; $Chi^2 = 9.95$, df = 2 (P = 0.007); $I^2 = 80\%$
Test for overall effect: Z = 0.88 (P = 0.38)

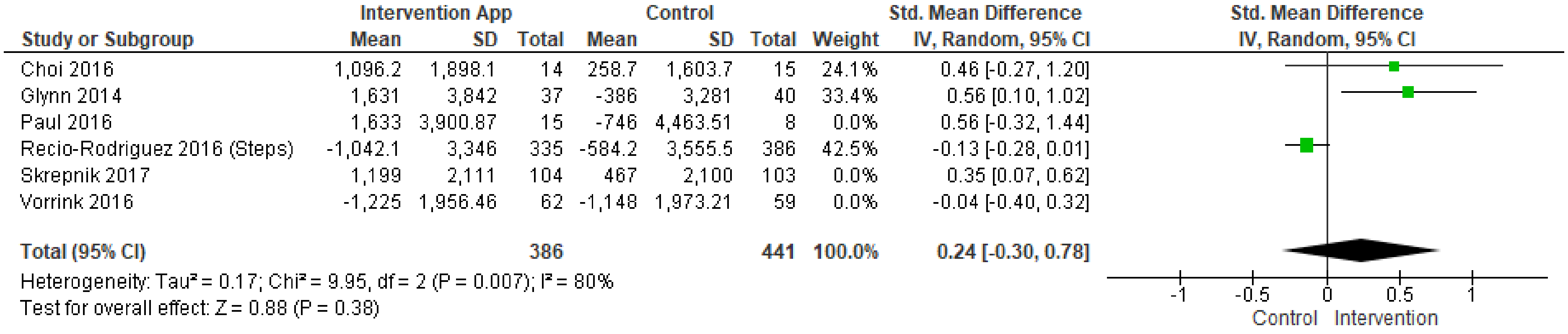

Figure 4: Standardised mean difference for apps targeting populations with specific health conditions.

Study or Subgroup	Intervention App Mean	Intervention App SD	Intervention App Total	Control Mean	Control SD	Control Total	Weight	Std. Mean Difference IV, Random, 95% CI
Choi 2016	1,096.2	1,898.1	14	258.7	1,603.7	15	0.0%	0.46 [-0.27, 1.20]
Glynn 2014	1,631	3,842	37	-386	3,281	40	0.0%	0.56 [0.10, 1.02]
Paul 2016	1,633	3,900.87	15	-746	4,463.51	8	11.0%	0.56 [-0.32, 1.44]
Recio-Rodriguez 2016 (Steps)	-1,042.1	3,346	335	-584.2	3,555.5	386	0.0%	-0.13 [-0.28, 0.01]
Skrepnik 2017	1,199	2,111	104	467	2,100	103	49.7%	0.35 [0.07, 0.62]
Vorrink 2016	-1,225	1,956.46	62	-1,148	1,973.21	59	39.3%	-0.04 [-0.40, 0.32]
**Total (95% CI)**			**181**			**170**	**100.0%**	**0.22 [-0.09, 0.53]**

Heterogeneity: $Tau^2 = 0.03$; $Chi^2 = 3.42$, df = 2 (P = 0.18); $I^2 = 41\%$
Test for overall effect: Z = 1.37 (P = 0.17)

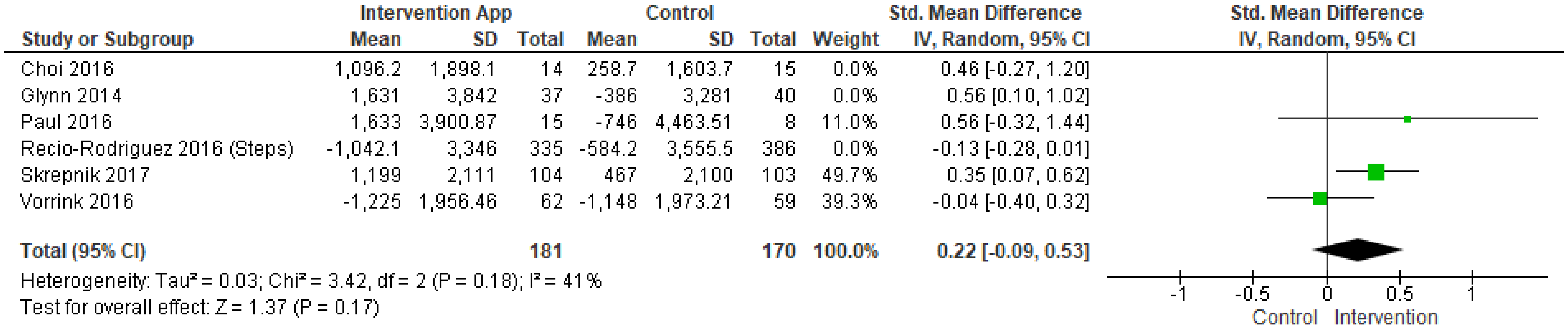

Supplement: Multimedia Appendix 4 [file jmir_v21i3e12053_app4.pdf]
